# Inbreeding and selection shape genomic diversity in captive populations: Implications for the conservation of endangered species

**DOI:** 10.1371/journal.pone.0175996

**Published:** 2017-04-19

**Authors:** Janna R. Willoughby, Jamie A. Ivy, Robert C. Lacy, Jacqueline M. Doyle, J. Andrew DeWoody

**Affiliations:** 1 Department of Forestry and Natural Resources, Purdue University, West Lafayette, Indiana, United States of America; 2 Department of Biological Sciences, Purdue University, West Lafayette, Indiana, United States of America; 3 San Diego Zoo Global Collections Department, San Diego, California, United States of America; 4 Chicago Zoological Society, Brookfield, Illinois, United States of America; 5 Department of Biological Sciences, Towson University, Towson, Maryland, United States of America; University of British Columbia Okanagan, CANADA

## Abstract

Captive breeding programs are often initiated to prevent species extinction until reintroduction into the wild can occur. However, the evolution of captive populations via inbreeding, drift, and selection can impair fitness, compromising reintroduction programs. To better understand the evolutionary response of species bred in captivity, we used nearly 5500 single nucleotide polymorphisms (SNPs) in populations of white-footed mice (*Peromyscus leucopus*) to measure the impact of breeding regimes on genomic diversity. We bred mice in captivity for 20 generations using two replicates of three protocols: random mating (RAN), selection for docile behaviors (DOC), and minimizing mean kinship (MK). The MK protocol most effectively retained genomic diversity and reduced the effects of selection. Additionally, genomic diversity was significantly related to fitness, as assessed with pedigrees and SNPs supported with genomic sequence data. Because captive-born individuals are often less fit in wild settings compared to wild-born individuals, captive-estimated fitness correlations likely underestimate the effects in wild populations. Therefore, minimizing inbreeding and selection in captive populations is critical to increasing the probability of releasing fit individuals into the wild.

## Introduction

The goal of many captive breeding programs is to retain the genetic/genomic diversity (GD) present in the captive founding population until such time that reintroduction of captive populations to wild habitats can occur [1, 2]. Retaining GD in a small, closed captive population is important as decreased GD across the genome often results in decreased fitness [3, 4]. Additionally, declines in GD can reduce evolutionary fitness and initiate a negative feedback loop that leads to smaller population sizes, more genetic drift, and increased inbreeding [5]. Furthermore, the loss of GD has been associated with diminished capacity to respond to environmental change and a reduction in the population growth rate [6, 7]. These are all significant concerns for populations that are destined for reintroduction in order to help establish self-sustaining wild populations.

In addition to the well-known impacts of inbreeding and genetic drift, selection can also rapidly deplete critical GD [8]. Relaxed natural selection (e.g., reduced competition and predation) via unintentional domestication can act to rapidly deplete GD in captive populations [9]. Furthermore, relaxed natural selection may lead to an accumulation of deleterious mutations [10], although this can be at least partially mitigated by captive population size and species generation time [11]. Adaptation to captivity is unlikely to significantly decrease the fitness of a captive population under active management, but such evolutionary change may have deleterious effects on individuals released into the wild [12, 9]. Therefore, understanding the response of GD to selection may suggest methods for minimizing adaptation to captivity in cases where supplementation of wild populations is the goal [13].

A reduction in GD, whether from inbreeding, drift, and/or selection, is often related to a reduction in fitness; this phenomenon is referred to as a heterozygosity-fitness correlation (HFC; see [4] or [14]). Although it would be desirable to retain 100% of the founding GD in a captive population, in practice this is difficult if not impossible. By comparing the relationship between markers and fitness, it may be possible to provide critical insights for population management by determining the mechanisms underlying HFCs [3]. For example, if heterozygosity calculated from a deep pedigree had a stronger correlation to fitness than did a small number of gene-associated single nucleotide polymorphisms (SNPs), the data would point towards multilocus genomic effects underlying the HFC (e.g., many genes of small effect). However, if SNPs located within or near genes under selection correlate better than the pedigree or a genome-wide SNP panel, the HFC would likely be due to selection on the genes that were genotyped, and this might suggest that targeting genome-wide retention of GD is important in light of GD at particular loci (e.g., major immune genes).

Breeding species in captivity in ways that minimize GD loss and adaptation to the captive environment is costly and complicated. However, recent efforts to experimentally measure evolution in captive populations have provided critical insight into management [15–16]. In one long running experiment, six populations of *Peromyscus leucopus*, all of which were started from the same 20 wild-caught founders, were bred for 20 generations using one of three commonly used breeding protocols: minimizing mean kinship to retain maximum GD, selection against particular stereotypic behaviors thought to be associated with docility, and random mating [15]. Subsequent analyses of these populations showed that minimizing mean kinship resulted in reduced inbreeding measured via the pedigree relative to random mating [15–16], which was consistent with the pedigree-estimated mean effective population size (minimizing mean kinship = 40.6, random mating = 28.2, docility selection = 24.0) [15]. However, the inter-protocol differences had a relatively small effect on GD measured via a panel of microsatellites or on the retention of mtDNA diversity [16]. Additional analyses showed selection against the docility-related behaviors was effective on one behavior (flipping) but not on another (gnawing), and that reproduction declined in populations where selection against these behaviors occurred. However, in populations where behaviors were not selected against, flipping increased; this was accompanied by an increase in the proportion of pairs breeding and a decrease in the time to conception [15]. Importantly, litter size, pup survival, and mass at weaning declined as inbreeding increased even as the number of breeding pairs increased and time to conception decreased. Thus, these populations demonstrate how rapidly populations can adapt to captivity, and how these changes can affect reproduction. However, the genomic consequences of the different breeding protocols and their associated changes have not yet been characterized but are of concern for managing captive populations.

Our current study had three primary objectives. First, we used a genome-wide SNP panel and six captive populations to evaluate how GD changes in light of three different breeding schemes that are often used in conservation efforts [15]. We hypothesized that inbreeding, drift, and selection would be mitigated most effectively in the populations bred to minimize mean kinship (MK), less effectively under random breeding (RAN), and least effectively in populations under artificial selection for docility (DOC). The MK protocol should minimize loss of lineages (and their associated GD) due to genetic drift, whereas the DOC protocol should lead to selective sweeps that deplete variation on chromosomes which contain genes associated with docility traits. Our second objective was to better understand the temporal trends in GD at nonneutral loci relative to changes that occur at neutral loci. We used population simulations to identify SNPs under selection and ultimately interpret the observed GD patterns in terms of loss due to inbreeding, drift, and selection. Our third objective was to evaluate HFCs in the context of a captive population. We did so by quantifying the relationship between fitness and GD as measured by pedigrees, as well as between fitness and GD as measured by genome-wide SNPs. Finally, we interpret our results in light of forces that alter GD in captive populations, namely inbreeding, drift, and artificial selection, to help conservationists more effectively retain GD for reintroduction efforts.

## Materials and methods

### Ethics statement

The breeding study and animal care protocols were approved by the Institutional Animal Care and Use Committee of the Chicago Zoological Society. Mice were collected from the wild under Scientific Permit W01.0845 from the Illinois Department of Natural Resources.

### Captive population management

The complete protocol and history of our captive populations is available elsewhere [15]. In summary, all captive colonies were founded with the offspring of 20 white-footed mice trapped in Volo Bog State Natural Area (Illinois) in 2001. Offspring from each of the founding pairs were divided equally into six breeding groups. Starting with the first captive born individuals, 20 pairs of mice from each group were chosen each generation for mating following one of three breeding protocols, which were chosen to mimic the breeding schemes commonly used for zoo populations. The breeding protocols included: RAN, MK, and DOC. In the RAN protocol, mice were selected randomly but the breeding of close relatives (i.e., those more closely related than the average pairwise kinship in the population at a given time) was avoided. The MK protocol selected individuals with the lowest, pedigree-calculated mean kinship values, and in theory, maximized the GD retained using a ranked MK procedure [17]. Individual mean kinship values were first calculated, individuals with the least desirable (i.e., highest mean kinship) values were removed from the list, kinships were recalculated, and this procedure continued until the list was exhausted. The last 20 males and 20 females (those with the lowest mean kinships) were selected as breeders for the next generation. Finally, the DOC protocol utilized stereotypic behaviors (as measured by time spent gnawing at cage bars and flipping at night) as indicators of docility. In other words, more sedentary individuals were assumed to be more docile relative to individuals engaging more frequently in stereotypic behaviors. By ranking activity level and choosing the most sedentary animals as breeders, individuals were paired to imitate the selection for docility that often occurs in captive breeding programs. Importantly, stereotypic behaviors were measured in individuals from all protocols. As in the RAN protocol, the MK and DOC protocols both avoided close inbreeding [as in 15]. In total, we maintained six populations (two replicates for each protocol) for up to 20 generations in captivity (the second DOC replicate went extinct after nine generations due to low reproductive success). Pedigrees were maintained in an Access (Microsoft Corp.) database and pedigree calculations that took place during the propagation of the captive populations were performed with PMx [17].

### SNP genotyping

We used a genotyping-by-sequencing approach in order to efficiently identify SNPs and genotype individuals [18]. This method utilizes restriction enzymes to reduce genome complexity, while efficiently tagging samples for individual identification during bioinformatic analysis. We followed [18], and our samples were digested (using *Pst*I), uniquely barcoded, and sequenced at the Genomic Diversity Facility at Cornell (http://www.biotech.cornell.edu/brc/genomic-diversity-facility). We genotyped individuals from each of the six captive populations, including approximately 15 individuals from the sixth, twelfth, and nineteenth generations. We also typed 2–4 offspring from each of the ten founding pairs as well as 15 individuals collected from the original wild source population in 2012.

Using the Uneak pipeline contained within the program Tassel [19–20] we simultaneously identified SNPs within the randomly sequenced tags and assigned individual genotypes. The Uneak pipeline works by first trimming reads to 64 base pairs and collapsing identical reads into tags. Next, candidate SNPs are identified by tags with single nucleotide differences, and these differences are organized into networks that connect multiple SNPs between tags containing similar sequences. Next, tags with much fewer reads compared to adjacent tags are removed, and the network edges connecting these tags are sheared, resulting in smaller SNP networks. Finally, only networks containing reciprocal pairs (i.e. only two read states) are kept for further analysis. We assumed an error rate of 0.03, which is a conservative estimate based on Illumina sequencing technologies [20]. Once identified via the Uneak pipeline, we filtered the raw SNP data to include only loci that were sequenced in 95% or more of the targeted individuals, and filtered individuals to include only those genotyped at >50% of the SNP loci. By analyzing samples from all populations and generation simultaneously, we attempted to reduce any ascertainment bias. However, ascertainment bias may occur when SNP loci are fixed for different alleles in groups with vastly different sample sizes, or when the minor allele is different between groups. Although some loci may not be scored due to low coverage in the most genetically divergent groups, the Uneak pipeline should result in relatively unbiased estimates of GD for our final set of SNPs.

### Tests of linkage and neutrality

In order to obtain unbiased estimates of population GD, we needed to ensure that our SNPs were independent. Using the ‘ld’ function in the snpStats package [21], we estimated D’ and grouped SNPs that had pairwise D’ estimates > 0.8. From these putative linkage groups, we then eliminated any SNP that occurred in more than one group. This process resulted in two sets of SNPs, singletons and those assembled into putative linkage groups, which were ultimately analyzed collectively by randomly selecting a single SNP from within the linkage groups and permuting our statistical models.

We also sought to identify SNPs under selection, and did so using simulations in R. We modified a computer program designed to mimic the breeder-selection protocols (i.e. MK, RAN, and DOC) to use SNP data [16] and simulated the expected change in allele frequencies for each SNP locus over 20 generations assuming no selection. We could not generate SNP genotypes for the original founders because of DNA degradation, so we began all of our simulations with the genotypes of founder offspring. Briefly, our program works by drawing on the data available from the actual breeding programs. At each generation, we selected breeders following the captive breeding protocols. In MK populations breeders were selected by minimizing mean kinship within the population whereas in RAN populations breeders were chosen randomly. Because we do not know the genetic underpinnings of the traits selected against in the DOC lines, we used a simple, additive genetic model with biallelic loci that were passed from parent to offspring. We determined the number of simulated offspring using data collected from the captive populations, specific to each breeding protocol; we randomly sampled the number of offspring produced by each simulated parent pair from the observed distribution of offspring successfully weaned by parent pairs in the captive populations. To generate offspring genotypes, we randomly selected one allele at each locus from each parent. Finally, we assigned male/female with a probability of 0.5 for either sex. A more detailed explanation of our simulation process is available in [16].

At each simulated generation, we calculated allele frequencies. In order to insure that we compared the same allele over all generations, we calculated (at each generation) the allele frequency only for the allele identified as minor in the empirical founder genotypes. After 100 replicate runs, we compared the empirical allele frequency to the distribution of simulated frequencies, and calculated a p-value for each SNP as the proportion of simulated replicate frequencies that were more extreme (i.e. closer to either 0 or 1) than the empirical allele frequency. We adjusted these p-values to account for the false discovery rate using the Benjamini and Hochberg [22] correction (p.adjust; R Develoment Core Team 2014) and identified SNPs with adjusted p-values < 0.05 as those likely impacted by selection because they were statistically inconsistent with neutral expectations (i.e., drift). We refer to such SNPs as "nonneutral". Although we simulated each SNP independently regardless of linkage group assignment, for our analyses we grouped results into putative linkage groups, and report the sum of the number of singletons and putative groups identified as nonneutral (i.e., under selection).

### Genome sequencing and annotation

As a method for assigning putative function to our SNPs, we performed genome sequencing and annotation. We sequenced a single wild *P*. *leucopus* individual captured from the locale that served as the source for our captive populations. We generated sequence data using two lanes of paired-end sequencing (read lengths of 100 bp) using an Illumina HiSeq2000 and cleaned the resulting reads (i.e. removed adaptor sequences, discarded reads <50 bp, and trimmed bases with Illumina Q-value ≤ 20) using Trimmomatic [23]. In order to generate scaffolds suitable for annotation, we combined *de novo* assembly with a reference guided approach. We used ABySS to generate our *de novo* assembly [24], and used BWA-SW [25] and the draft *P*. *maniculatus* assembly (NCBI assembly accession GCA_000500345.1) to improve our *de novo* assembly [26]. We used MAKER [27] to annotate the scaffolds by 1) generating *ab initio* predictions with SNAP [28] and 2) using *P*. *maniculatus* EST libraries [29] and *Mus musculus* protein sequences downloaded from the UniProtKB database (http://www.uniprot.org/uniprot/) to provide support for a subset of these predictions. We additionally used InterProScan to identify putative protein domains and provide support for *ab initio* predictions. Finally, we used Bowtie2 to map our SNP sequences onto the annotated scaffolds [30] and used Pfam and InterPro to tie gene functions to particular SNPs [31, 32].

### Effects of inbreeding, drift, and selection

We compared the impact of the three captive breeding protocols on GD using our SNP genotypes. We analyzed the SNPs using three data partitions: 1) all SNPs; 2) only SNPs identified as impacted by selection for a particular breeding protocol (i.e., nonneutral SNPs); 3) only SNPs not identified as impacted by selection for a particular protocol (i.e., neutral SNPs). For each individual within each population at each generation, we calculated the average multilocus heterozygosity (MLH; R package snpStats) [21] as a measure of both variability within an individual and, when averaged across a group, an estimate of GD within the group. We calculated individual MLH estimates by averaging (across 100 replicates) MLH at all singleton SNPs and one randomly selected SNP from each of our putative linkage groups. Additionally, we calculated F, which is the probability of a locus being identical by descent [33] using the pedigrees and the calcInbreeding function (R package pedigree) [34], averaged across all individuals in population at each generation.

We evaluated the long-term impact of breeding protocol on GD using our estimates of genome-wide diversity. We compared the rate of change in average MLH by comparing the means ± SE, averaged across individuals at each generation for each population. We compared our estimates of GD calculated from SNPs (MLH) and the pedigrees (F) using a Spearman correlation, then permuted the SNP estimates to calculate a p-value for each comparison.

We analyzed the effect of selection across protocols and generations using two approaches. In order to understand how the number of SNPs under selection differed between protocols, we first compared the total number of nonneutral SNPs across populations and generations using a 2-way ANOVA, and analyzed the direction of differences using a post-hoc Tukey test in R. We then sought to understand the effects of selection within an individuals’ genome. Therefore, we then analyzed the difference in MLH between neutral and nonneutral SNPs by calculating mean differences between the estimates for each individual. We compared the mean difference across individuals within each protocol using the standard error (SE) estimates around each.

### Heterozygosity-fitness correlations

We examined the relationship between fitness and GD using a) the pedigree and b) the SNP data. We utilized our MLH estimates from the SNP genotypes and we used the inbreeding coefficient (F) from the pedigrees. We used an individual’s mass at weaning and the total number of offspring weaned as putative fitness estimates. Additionally, we compared three morphological measurements to GD: ear size, hind foot length, and tail length. We determined the relationship between our GD estimates for all individuals and for males and females separately across all of our replicate populations using a Spearman correlation. We used 1000 permutations over our GD estimates to estimate a p-value, and considered a fitness correlation significant only if it had a p-value < 0.05.

## Results

### SNP genotyping and tests of linkage and neutrality

We attempted genotyping-by-sequencing [18] for a total of 285 individuals from six captive lines across four generations (captive generations 1, 6, 12, and 19) as well as the wild source population. After quality filtering and calling via the Uneak pipeline [19–20], our final dataset consisted of 5454 SNPs genotyped across at least 95% of 273 samples (S1 File, Data Dryad doi:10.5061/dryad.7pt2m). These SNPs included 705 singletons (unlinked to any other SNPs) as well as 4749 SNPs distributed among 531 putative linkage groups, which each contained from 2–35 SNPs (mean of 2.5). For all subsequent population GD estimates, we used all singleton SNPs and one linked SNP randomly pulled from each linkage group, averaged over 100 replicates.

We identified nonneutral SNPs by comparing simulated allele frequency distributions under a null model of neutrality to our empirical allele frequencies (S1 Table). Across all populations and all generations, we identified a grand mean of 171 unique SNPs under selection. On average, 11% of nonneutral SNPs identified were shared across all sampled generations within a population, although more were shared from generation 6 to 12 (31%) and from 12 to 19 (42%). We found an average of 21 nonneutral SNPs that occurred within each sampled generation in the populations genotyped at multiple time points (S1 Table). In total, three nonneutral, singleton SNPs were annotated (DOC: TP77060; RAN: TP161428, TP75443), all with fairly common functions (i.e. protein kinase domain, myosin-binding motif of peroxisomes, zinc finger).

### Genome sequencing and annotation

We successfully generated 2 lanes of Illumina HiSeq2000 genomic sequence data. Because we were working with a non-model species and lacked significant genomic resources, we attempted to improve our *de novo* assembly by leveraging a reference assembly. Our assembly contained 2,575,218 Kbp, including 64,3706 scaffolds with our longest scaffold being 79,125 bp and an N50 of 4087 bp. We also generated 30,269 unique *ab initio* gene predictions, 3905 of which were supported with EST, protein, or InterPro evidence. (Reads have all been deposited into NCBI, accession PRJNA375113 and assembly and annotation have been archived in Data Dryad doi:10.5061/dryad.7pt2m.) In total, 68 of our SNPs mapped to within 10,000 bp of an annotated locus, 41 of which were located within a coding region (S1 Table).

### Effects of inbreeding, drift, and selection

**Fig 1:**
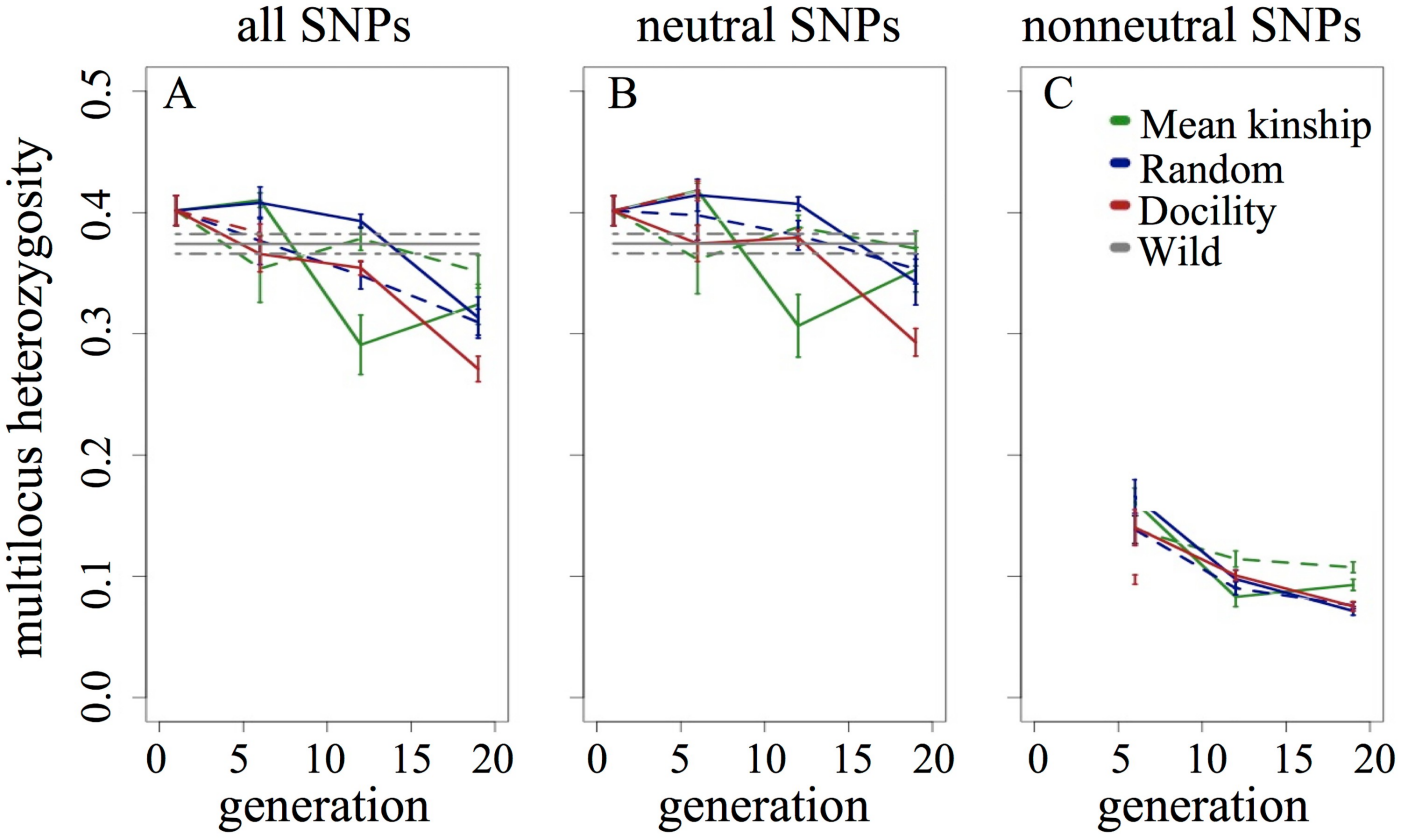
Effects of inbreeding, drift, and selection in captive populations. Mean multilocus heterozygosity (MLH) for six captive populations and the wild source population. Replicate populations are shown by solid (replicate 1) and dashed (replicate 2) lines. Estimates are shown for all genotyped SNPs (A), neutral SNPs (B) and nonneutral SNPs (C) as determined via simulations. Error bars represent SE calculated across individual estimates.

We compared MLH at all SNPs, neutral SNPs, and nonneutral SNPs for each population and generation then assessed significance by comparing SE estimates around each mean. For all three SNP data partitions, the difference in MLH between breeding protocols was relatively small, although we did find that the MK populations had more GD in nonneutral SNPs by generation 19 (Fig 1A–1C). The SNP heterozygosity estimates for the entire SNP dataset from generations 1, 6, and 12 for all captive populations overlapped with the estimates from the wild source population, with estimates from generation 19 being notably reduced. [We did not make this comparison for nonneutral SNP groups because we could not realistically simulate selection in the wild population.] However, nonneutral SNPs had a much lower MLH and, at least for the RAN and DOC populations, MLH decreased at each generation. The MK populations exhibited a slightly different pattern; between generations 12 and 19, heterozygosity increased in replicate 1 but showed no change in replicate 2 (Fig 1C).

**Fig 2:**
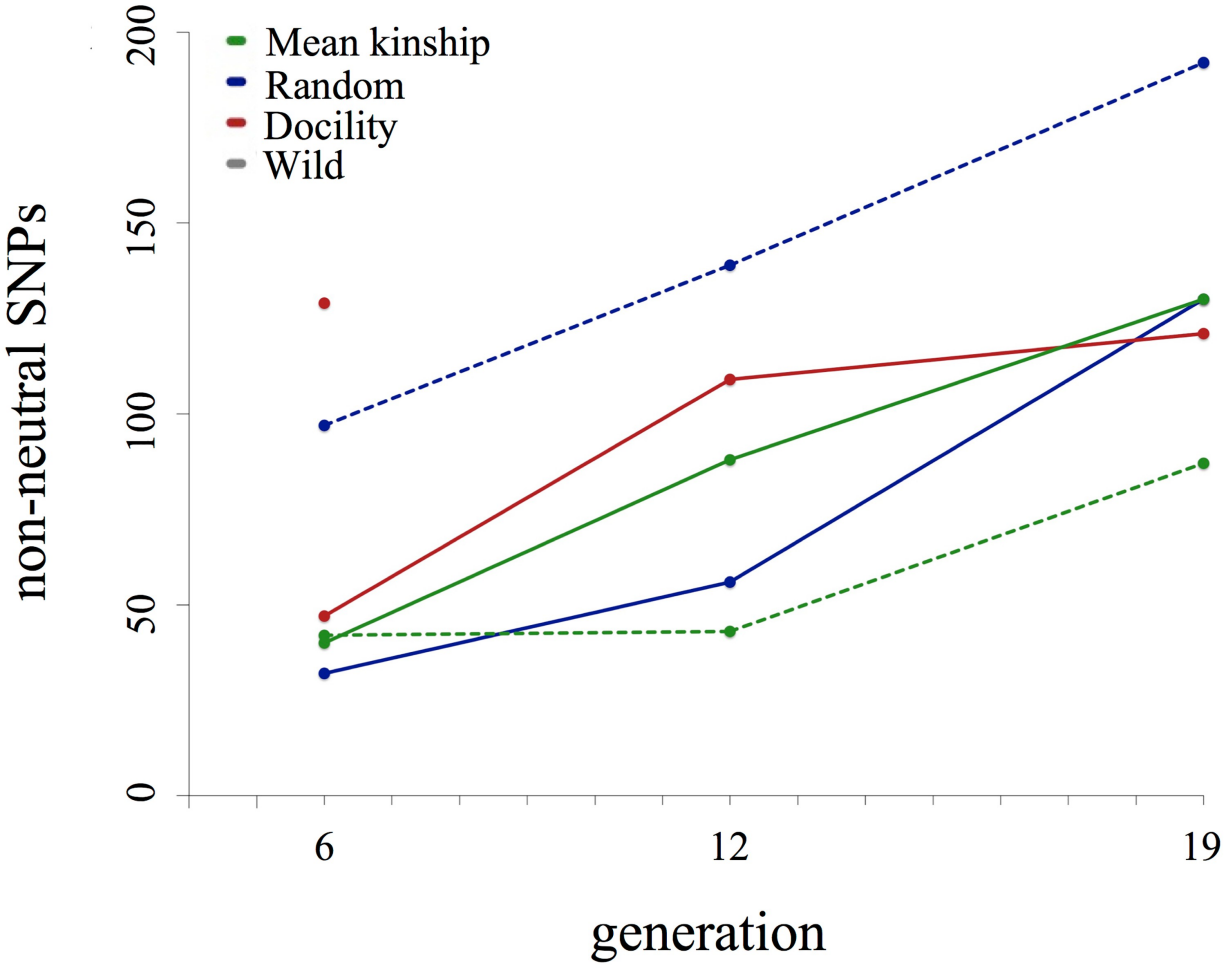
Comparison of the number of nonneutral SNPs detected per population at generations 6, 12, and 19. Replicate populations are shown by solid (replicate 1) and dashed (replicate 2) lines. Although no significant difference was detected between different breeding protocols (ANOVA: F = 4.8037, df = 2, p = 0.19), the number of nonneutral SNPs identified per generation was significantly higher in generation 19 compared to generation 6 (ANOVA: F = 1.911, df = 2; Tukey: g19-g6: diff = 67.5, adjusted p = 0.026).

**Fig 3:**
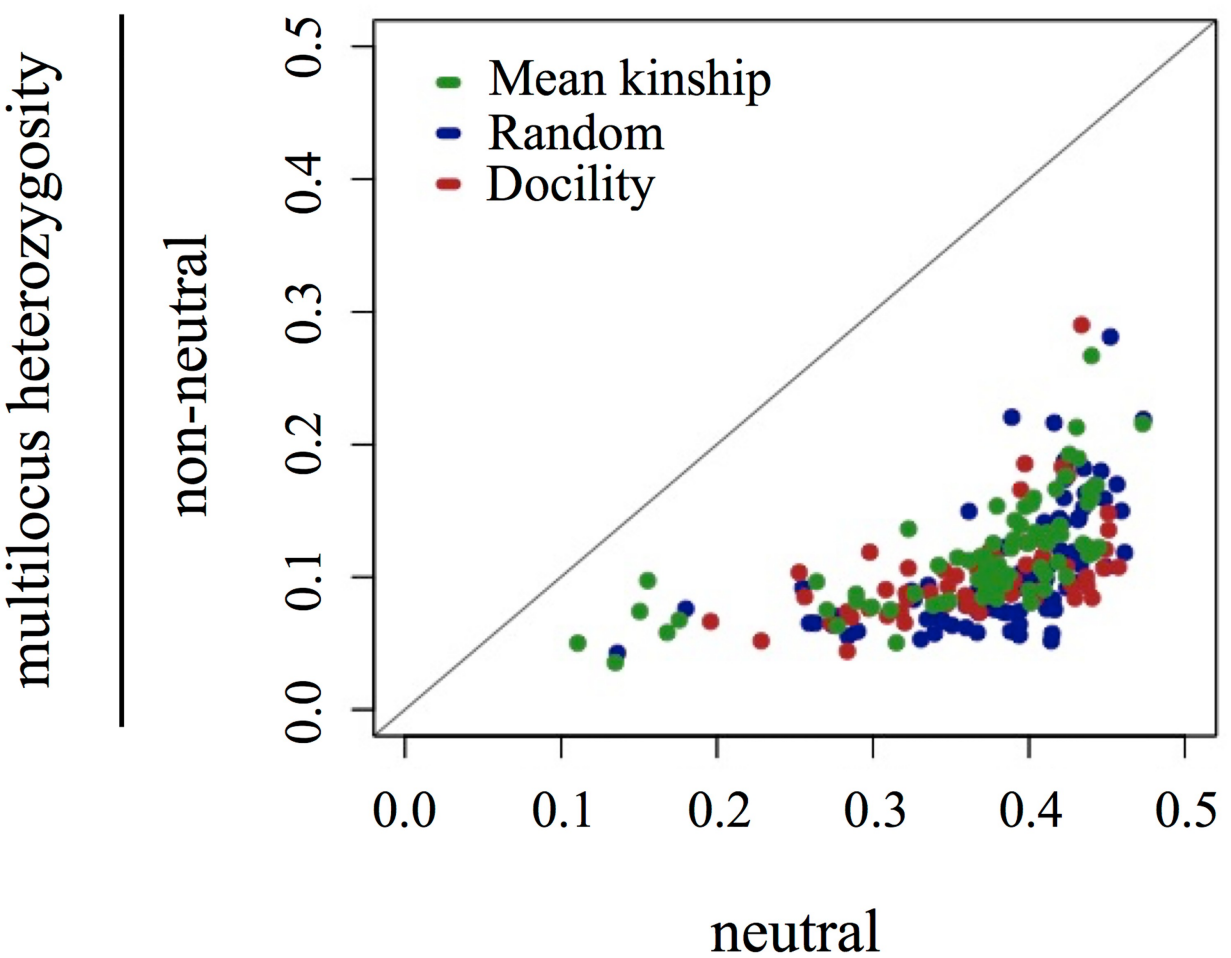
Within-individual estimates of multilocus heterozygosity (MLH) calculated using neutral as well as nonneutral SNPs. Diagonal line illustrates location of perfect concordance between neutral SNPs and non-neutral SNPs; individuals with equal MLH in neutral and nonneutral SNPs would be plotted on the diagonal. Overall, nonneutral SNPs have less diversity (lower MLH) compared to estimates calculated from neutral SNPs.

In all populations, the number of SNPs identified as nonneutral increased across generations (Fig 2), although the effects of selection were not entirely consistent across generations or populations. Our ANOVA results suggested that the number of nonneutral SNPs was not different between protocols (F = 4.8037, df = 2, p = 0.19), but that generation was significantly related to number of nonneutral SNPs (F = 1.911, df = 2, p = 0.03). Specifically, selection increased over generations, as generation 19 had significantly more nonneutral SNPs than generation 6 (Tukey test results: g19-g6 diff = 67.5, adjusted p = 0.026; g12-g6 diff = 22.5, adjusted p = 0.578; g19-g12 diff = 45.0, adjusted p = 0.168). The increase in the number of nonneutral SNPs across generations may well have been due to adaptive changes across the genome, but may also indicate that the effects of selection became increasingly detectable over time as the allele frequencies became more extreme relative to the expected distribution of frequencies. For example, weak selection on a SNP in generation 6 may not have been detectable until generation 12 because the change in allele frequency was small but, by generation 12, selection was detectable. The effects of selection were consistent across protocols in that all individuals had reduced MLH in the nonneutral SNPs compared to neutral SNPs (Fig 3). However, we found that the effects of selection on measures of GD varied by protocol: the difference between MLH estimates for individuals using nonneutral SNPs compared to neutral SNPs were significantly higher in the RAN populations compared to estimates from the MK protocol (Table 1).

**Table 1:** Mean difference of within individual estimates of heterozygosity (MLH) between nonneutral and neutral SNPs. Bolded values are significantly different from each other. Differences between nonneutral and neutral SNPs in MLH were significantly smaller in the minimizing mean kinship populations compared to the randomly mating population.

Protocol	Number of individuals	Mean difference	Standard error interval
Random	87	0.276	**0.266, 0.287**
Docility	60	0.264	0.251, 0.278
Mean kinship	81	0.252	**0.239, 0.264**

Finally, we compared the genome diversity estimates provided by the molecular markers with those of the pedigrees. Within each breeding protocol, we estimated MLH via SNP genotypes and estimated F from the pedigree. We found that MLH was best correlated to F in DOC, and moderately well correlated to F in RAN and MK (S3).

### Heterozygosity-fitness correlations

We examined the relationship between individual fitness and GD, again using MLH and F. Across all of the SNP-calculated fitness correlations, several correlations were positively significant; heterozygosity estimated from nonneutral SNPs was correlated to weight at weaning, as was heterozygosity estimated from all SNPs in males (Table 2). In females, tail length was correlated to heterozygosity as measured with all SNPs and with only neutral SNPs. In males, tail length was only correlated with the all SNP-estimated heterozygosity. The pedigree calculated F was significantly negatively correlated with weight at weaning, tail length, and ear size in females and males, and hind foot length in females only (Table 3).

**Table 2:** Spearman correlations (r) between genetic diversity (MLH) and trait measures. The p-values, shown in parentheses, were calculated via 1000 permutations. We estimated correlations using three groups of SNPs: all SNPs, nonneutral SNPs, and neutral SNPs. Significant values, identified by p-values < 0.05, are shown in bold. Italicized traits indicate traits most tightly associated with fitness.

	females	males
*total number offspring weaned*		
all SNPs	0.125 (0.093)	0.068 (0.254)
nonneutral SNPs	**0.173 (0.039)**	**0.193 (0.039)**
neutral SNPs	0.125 (0.120)	0.045 (0.320)
*weight at weaning*		
all SNPs	0.099 (0.133)	**0.153 (0.043)**
nonneutral SNPs	**0.161 (0.026)**	**0.234 (0.007)**
neutral SNPs	0.099 (0.139)	0.092 (0.153)
tail length		
all SNPs	**0.161 (0.039)**	**0.195 (0.017)**
nonneutral SNPs	0.142 (0.068)	0.080 (0.208)
neutral SNPs	**0.161 (0.040)**	0.179 (0.312)
ear size		
all SNPs	0.028 (0.412)	0.097 (0.142)
nonneutral SNPs	0.091 (0.162)	**0.197 (0.022)**
neutral SNPs	0.028 (0.397)	0.031 (0.375)
hind foot length		
all SNPs	0.109 (0.107)	0.027 (0.392)
nonneutral SNPs	0.136 (0.072)	0.100 (0.163)
neutral SNPs	0.109 (0.098)	0.040 (0.348)

**Table 3:** Spearman fitness correlations (r) between pedigree-based inbreeding and trait measures. The p-value, shown in parentheses, was calculated via 1000 permutations. We estimated genome-wide diversity using the inbreeding coefficient (F) as calculated from the pedigree. Note that F is inversely related to heterozygosity. Significant values, identified by p-values < 0.05 are shown in bold. Italicized traits indicate traits most tightly associated with fitness.

	Females	Males
*Total number offspring weaned*	-0.144 (0.072)	-0.167 (0.050)
*Weight at weaning*	**-0.286 (<0.001)**	**-0.341 (<0.001)**
Tail length	**-0.154 (0.044)**	**-0.245 (0.006)**
Ear size	**-0.226 (0.003)**	**-0.282 (0.001)**
Hind foot length	**-0.164 (0.037)**	-0.068 (0.231)

## Discussion

The maintenance of genetic diversity (GD) in managed populations is an important goal of modern conservation [35], but captive breeding programs face inherent challenges associated with small population sizes, limited gene flow, and artificial environments. These challenges have the potential to significantly change the evolutionary trajectory of a given gene pool in a manner that may ultimately decrease population fitness relative to wild progenitors. Our study directly characterizes and quantifies such changes in GD across time, across breeding protocols, across marker type (i.e., neutral vs. nonneutral), and across sources of genetic data (molecular vs. pedigree). We hypothesized that GD should be retained best in the MK populations and worst in the DOC populations, due to reduced inbreeding and selection under the MK breeding protocol that aims to reduce loss of genetic lineages. Although the quantity of nonneutral SNPs was not different between protocols (S2 Table), the effect of selection (i.e., comparing selected to neutral SNPs) on GD was reduced in MK relative to RAN (Table 1). Finally, we found that GD was generally related to fitness (Tables 2 and 3).

### Effects of inbreeding, drift, and selection

We hypothesized that the three breeding protocols would retain GD with varying efficiency, based on empirical data derived from populations of *Drosophila* [36–38] and previous theoretical work [39]. Previous analysis of the pedigrees for our populations indicated that each breeding protocol had significantly different evolutionary trajectories [16]. However, vagaries associated with Mendelian inheritance cast doubt on the characterization of genome-wide diversity solely from pedigree estimates [40]. Similar to our earlier microsatellite results, the difference between captive populations in multilocus heterozygosity calculated from the SNP genotypes was expectedly small (Fig 1A–1C). Although the MK protocol reduced inbreeding as measured by the pedigree [15–16], SNP-estimated GD was still lost at a rate similar to random mating, potentially due to the inability to completely prevent drift and selection by means of the breeding protocol. Similarly, the previously estimated rate of loss of GD measured via microsatellites and mtDNA haplotypes was comparable in the RAN and MK lines [16]. This is supported by estimates of effective population size (N_e_), generated from our final estimates of heterozygosity. Assuming the founders represented the initial heterozygosity present in all populations [41], we estimated that the final, mean effective population size was largest in the MK populations (115.8), followed by RAN (75.3) and DOC (48.8) populations. Although drift occurs randomly, the relationship between selection and heterozygosity could be explained by selective sweeps due to local or direct effects [36]. In either case, sweeps would remove variability and result in GD disparities between neutral and nonneutral SNPs that should increase over time.

Random genetic drift will nearly always be a problem for multi-generation captive programs due to small breeding populations, but reducing the effects of selection is an aspirational goal in captive breeding [2, 37, 42], particularly when retention of GD is the aim [38]. In our captive populations, we found that the number of detectably nonneutral SNPs increased over time (Fig 2 and S1 Table) and that MLH was significantly different between nonneutral SNPs and neutral SNPs (Fig 3). In nonneutral SNPs, MLH decreased relative to the neutral comparisons. Additionally, mean estimates were significantly different between populations by generation 19 and indicated that the MK population had retained more GD at SNPs under selection compared to both DOC and RAN populations (Fig 1). Although the number of selected SNPs did not differ between protocols (S2 Table), we suggest that the magnitude of the effect of selection was reduced in MK, as evidenced by the decreased change between neutral SNPs and nonneutral SNPs and observed in other populations managed in a similar manner [2, 38].

In contrast to the MK populations, selection should have been the strongest in the DOC protocols due to the selection procedure employed. However, the number of nonneutral SNPs was not different between protocols (S2 Table), and the MLH difference in nonneutral SNPs within individuals were not different between the DOC and MK protocols. Although this may be due to poorly characterized behavioral selection in our simulations (see methods), we suggest that this may be a result of relative strength of selection for the specific behavioral traits we measured (strong selection on a few loci with major effects on the traits) compared to general adaptation to captivity (weak selection on each of many loci throughout the genome). If relatively strong selection occurred on only a few of the SNPs under selection, the effect on GD across all nonneutral SNPs may not have been detectable.

In addition to the observed differences among breeding protocols, we also observed differences in GD within replicates. For example, the MK replicates had drastically different heterozygosity values in generation 12, but ended at similar values in generation 19 that were different from DOC and often RAN lines (Fig 1A–1C). Although we cannot identify the precise cause of this variation with our current dataset, these variations suggest that either A) the vagaries of Mendelian inheritance vary genome-wide diversity over a few generations and/or B) selection during captive breeding selects for behaviors at least partly controlled by different genes. Because the variance between replicates was smaller in our non-neutral SNPs, we suggest that inheritance differences, likely exacerbated by small population sizes and strong effects of drift, most likely led to the deviations we observed between replicates.

### Heterozygosity-fitness correlations

SNPs on a genomic scale are expected to provide an estimate of genome-diversity commensurate with those estimated from a pedigree [43]. However, genetic diversity estimated from markers may diverge from pedigree-derived measures of heterozygosity over many generations due to chance events associated with Mendelian segregation that are not captured in a pedigree [44]. In our comparison of SNP and pedigree estimated measures of GD, we found that weakest relationship (lowest spearman correlation coefficient) occurred in the MK populations ($\bar{r}$ = -0.419) compared to RAN and DOC ($\bar{r}$ = -0.611 and -0.681, respectively; S3 Table). Although not conclusive, this suggests that the build-up of Mendelian-associated errors may erode the usefulness of a pedigree. Because the pedigrees did not guide breeding decisions in the RAN and DOC populations, these protocols were less biased by this effect, although the pedigree and SNP genotypes were still not completely concordant (S3 Table).

Typically, the correlation between GD and traits linked to fitness is positive, but lacks power [14, 45]. We found that fitness was positively associated with increased MLH in most cases (S3 Table and Table 2). Additionally, most of our correlations (SNPs: females $\bar{r}$ = 0.12, males $\bar{r}$ = 0.11, combined $\bar{r}$ = 0.12; pedigrees: females $\bar{r}$ = 0.19, males $\bar{r}$ = 0.22, combined $\bar{r}$ = 0.21) were larger than the average HFC reported in surveys of animals (r ~0.05; 45), including captive populations (e.g., domesticated zebra finch, *Taeniopygia guttata*; $\bar{r}$ ~0.1; 46). The relatively large HFCs we observed were likely due to the increased inbreeding and associated inbreeding depression (i.e., manifestation of deleterious traits) in our study system. Somewhat surprisingly, we found that the total number of offspring weaned was not as strongly correlated to GD estimates as was the weight at weaning, even though offspring production provides a good approximation of true fitness [46–47]. Presumably, the reduced correlation between GD and number of offspring weaned may be due to the breeder selection protocols used in our captive populations. For example, producing multiple offspring in the MK lines did not necessarily lead to increased fitness in our captive populations because the breeder selection protocol was likely to select only a single offspring from a given pair. However, the evolutionary history of the captive populations–descended from wild mice a maximum of 19 generations prior to genotyping–would lead to a residual correlation between high GD and high fecundity [48]. Therefore, except in the RAN populations, increased fecundity did not necessarily increase fitness, likely weakening the HFCs we detected.

With a sufficiently deep and accurate pedigree, the pedigree-calculated inbreeding coefficient *F* should be the best predictor of fitness because a true pedigree should offer a better measure of genome-wide diversity than any panel of markers short of full genome sequencing [17]. Under the general effect hypothesis, whereby HFCs are due to genome-wide effects [3–4], we expect stronger correlations between fitness and GD at neutral SNPs compared to the correlation between fitness and GD at nonneutral SNPs because the latter may not influence the fitness traits measured and may represent only a small proportion of the underlying genetic variation. Under the local or direct effect hypotheses, whereby HFCs are due to selection on or near the molecular markers, we expect much stronger correlations between fitness and GD at nonneutral SNPs than we do between fitness and GD at neutral SNPs (Table 4). Our empirical data indicate that in measures most directly associated with fitness–total number of offspring weaned and weight at weaning–heterozygosity at nonneutral SNPs was strongly correlated. However, we also found that the pedigree F was weakly correlated with the number of offspring weaned and strongly correlated (the highest fitness correlation we observed) with weight at weaning (Tables 2 and 3), supporting the general effects hypothesis. The morphometric measures were typically equally correlated with the pedigree estimates of diversity, which is again consistent with the general effect hypothesis for those traits. We interpret these trends as evidence supporting the both the general effects hypotheses and the local/direct hypotheses. As we move ever towards true population genomics, it will become much easier to partition the variation in HFCs between genome-wide and locus-specific effects.

**Table 4:** Expectations regarding heterozygosity-fitness correlations (HFCs). Under the general effect hypothesis, HFCs are due to genome-wide effects that should result in stronger correlations between genetic diversity at neutral loci compared to nonneutral loci. In contrast, under both the local and direct effect hypotheses, HFCs should result from selection on or near the molecular markers themselves, in which case there should be stronger correlations between fitness and genetic diversity at nonneutral loci than between fitness and genetic diversity at neutral loci.

HFC mechanism hypothesis	Expected HFC pattern	Inference
general effect	r_neutral loci_ > r_nonneutral loci_	genome-wide heterosis
local or direct effect	r_nonneutral loci_ >> r_neutral loci_	selection on/near markers

Regardless of the mechanisms behind the HFCs, minimizing the effects of reduced genetic diversity on fitness by limiting evolution in captive environments is important when reintroduction is the goal. As populations adapt to captivity over generations, it is likely that captive-born individuals become less fit in wild settings compared to wild-born individuals [49], meaning that captive-estimated fitness correlations likely underestimate the effects of reduced genetic diversity in wild populations [50]. Therefore, minimizing the loss of genetic diversity in captive populations, with the ultimate goal of retaining characteristics required for survival and reproduction, is important for any conservation program aimed ultimately at releasing fit individuals into the wild.

## Conclusions

The maintenance of genetic diversity in managed populations is critical in providing the best possible chance of successful reintroduction. However, captive populations are inherently small, have limited gene flow, and exist in artificial environments, and these characteristics often significantly alter the evolutionary trajectory, decreasing population fitness relative to wild progenitors. Our data indicate that minimizing mean kinship among breeders reduces inbreeding compared to random mating or selection on a particular behavior and helps temper adaptation to the artificial environment. Furthermore, we found that genome-wide heterozygosity was also related to fitness. Therefore using protocols that minimize mean kinship should result in the production of individuals better able to survive in wild environments. Beyond applied implications for conservation breeding programs, our results also elucidate how genetic variation is distributed and maintained across the genome in the face of drift and selective processes.

## Supporting information

S1 Table Gene annotations for genotyping-by-sequencing identified SNPs. Pfam descriptions were collected from the protein family database, and gene ontology terms and codes for biological processes, molecular functions, and cellular components were identified using InterPro.
(DOCX)

S2 Table Number of nonneutral SNPs (i.e., those that are likely under selection). SNP counts are noted for each replicate protocol, at each generation and the number shared with all previous generations are noted in parentheses. The ‘Unique’ column indicates the number of unique SNPs within the row, whereas “Shared” indicates the number shared within the row. ANOVA indicated that there was no difference between the number of SNPs under selection between protocols (F = 4.8037, df = 2), but there was a significant difference between generations (F = 1.911, df = 2). Our post-hoc Tukey test suggests that the effects of selection increased over time, as generation 19 had significantly more SNPs compared to generation 6 (g19-g6: diff = 67.5, adjusted p = 0.026; g12-g6: diff = 22.5, adjusted p = 0.578; g19-g12: diff = 45.0, adjusted p = 0.168).
(DOCX)

S3 Table Comparison of SNP estimated genomic diversity (multilocus heterozygosity) to pedigree estimates of genetic diversity (F). We used Spearman correlation coefficients (r) and p-values were estimated via 1000 permutations (all p <0.001).
(DOCX)

S1 File Final, quality filtered SNPs used in analyses. DNA samples were taken across 6 populations (random 1, random 2, mean kinship 1, mean kinship 2, docility 1, docility 2) and 4 generations (1, 6, 9, 19) and subsequently digested (using Pstl) and sequenced at Cornell according to [18]. The raw data were scored using Uneak pipeline within Tassel [20]. Within the table, genotypes are represented by IUPAC nucleotide codes: C = C/C; G = G/G; T = T/T; M = A/C; R = A/G; W = A/T; S = C/G; Y = C/T; K = G/T; N = missing data.
(ZIP)
