# Supplementary material for: Inbreeding and selection shape genomic diversity in captive populations: Implications for the conservation of endangered species
Source: PLoS One. 2017 Apr 19;12(4):e0175996. doi: 10.1371/journal.pone.0175996 (PMC5396937; doi:10.1371/journal.pone.0175996)
Supplement: S1 Table — Pfam descriptions were collected from the protein family database, and gene ontology terms and codes for biological processes, molecular functions, and cellular components were identified using InterPro. (DOCX) [file pone.0175996.s001.docx]

S1. Gene annotations for genotyping-by-sequencing identified SNPs. Pfam descriptions were collected from the protein family database, and gene ontology terms and codes for biological processes, moleulcar functions, and cellular components were identified using InterPro.

| **SNP name** | **Pfam Description** | **Biological process code** | **Biological process** | **Molecular function code** | **Molecular function** | **Cellular component code** | **Cellular component** |
| --- | --- | --- | --- | --- | --- | --- | --- |
| TP21382 | 7 transmembrane receptor, Hormone receptor domain | 0007186 | G-protein coupled receptor signaling pathway | 0004930 | G-protein coupled receptor activity | 0016021 | integral component of membrane |
| TP89063 | 7 transmembrane receptor (Secretin family) | 0007186 | G-protein coupled receptor signaling pathway | 0004930 | G-protein coupled receptor activity | 0016021 | integral component of membrane |
| TP28471 | ATPase family associated with various cellular activities (AAA) |  |  | 0005524 | ATP binding |  |  |
| TP112325 | Low-density lipoprotein receptor domain class A |  |  | 0005515 | protein binding |  |  |
| TP81469 | Low-density lipoprotein receptor domain class A |  |  | 0005515 | protein binding |  |  |
| TP77060 | Protein kinase domain | 0006468 |  | 0005524, 0004672 | ATP binding, protein kinase activity |  |  |
| TP154679 | RNA recognition motif |  |  | 0003676 | nucleic acid binding |  |  |
| TP151417 | Zinc finger |  |  | 0046872 | metal ion binding |  |  |
| TP162555 | Zinc finger |  |  | 0046872 | metal ion binding |  |  |
| TP108696 | Class I Histocompatibility antigen, domains alpha 1 and 2 | 0006955, 0019882 | antigen processing and presentation, immune response |  |  |  |  |
| TP154040 | Carboxylesterase family |  |  |  |  |  |  |
| TP56215 | Carboxylesterase family |  |  |  |  |  |  |
| TP25937 | Helicase conserved C-terminal domain |  |  | 0005524, 0004386, 0003676 | helicase activity, nucleic acid binding |  |  |
| TP54789 | Helicase conserved C-terminal domain |  |  | 0003676, 0004386, 0005524 | nucleic acid binding, helicase activity, ATP binding |  |  |
| TP152192 | Ubiquitin carboxyl-terminal hydrolase | 0006511 | Ubiquitin-dependent protein catabolic process | 0036459 | ubiquitinyl hydrolase activity |  |  |
| TP1173 | Minichromosome maintenance, DNA replication | 0006260 | DNA replication | 0003677, 0005524 | DNA binding, ATP binding |  |  |
| TP142059 | Sterile alpha motif (or SAM) domain |  |  |  |  |  |  |
| TP76764 | SAM domain (Sterile alpha motif) |  |  |  |  |  |  |
| TP109491 | Cadherin cytoplasmic region | 007156 | homophilic cell adhesion via plasma membrane adhesion molecules | 0005509 | calcium ion binding | 0016020 | membrane |
| TP56014 | Cadherin cytoplasmic region | 0007156 | homophilic cell adhesion | 0005509 | calcium ion binding | 0016020 | membrane |
| TP83376 | Cadherin cytoplasmic region | 0007156 | homophilic cell adhesion | 0005509 | calcium ion binding | 0016020 | membrane |
| TP101973 | KRAB box, zinc fingers | 006355 | regulation of transcritpion, DNA-templated | 003676 | nucleic acid binding | 005622 | intracellular |
| TP75663 | Type I phosphodiesterase / nucleotide pyrophosphatase |  |  | 0003824 | catalytic activity |  |  |
| TP7144 | Spc97 / Spc98 family | 0000226 | microtubule cytoskeleton organization |  |  | 00005815, 0000922 | microtubule organiztion center, spindle pole |
| TP151488 | CS domain |  |  |  |  |  |  |
| TP31688 | Origin recognition complex | 0006260 | DNA replication | 0003677, 0005524 | DNA binding | 0005664 | nuclear origin of replication recognition complex |
| TP75346 | Origin recognition complex (ORC) subunit 3 N-terminus | 0006260 | DNA replication | 0003677 | DNA binding | 0005664 | nuclear origin of replication recognition complex |
| TP90934 | Dynein heavy chain, N-terminal region 2 |  |  |  |  |  |  |
| TP58627 | Putative transmembrane protein |  |  |  |  |  |  |
| TP102142 | Ankyrin repeat |  |  |  |  |  |  |
| TP148510 | Ankyrin repeat |  |  |  |  |  |  |
| TP75443 | Zinc-finger of C2H2 type |  |  |  |  |  |  |
| TP57019 | AAA domain |  |  |  |  |  |  |
| TP25188 | Olfactory receptor |  |  |  |  |  |  |
| TP75440 | Olfactory receptor |  |  |  |  |  |  |
| TP145184 | Leucine-rich repeat |  |  | 0005515 | protein binding |  |  |
| TP155494 | Leucine-rich repeat |  |  | 0005515 | protein binding |  |  |
| TP167804 | Leucine-rich repeat-containing protein 37 family |  |  |  |  |  |  |
| TP33396 | Leucine-rich repeat-containing protein 37 family |  |  |  |  |  |  |
| TP19532 | Domain of unknown function |  |  |  |  |  |  |
| TP20808 | Glycosyl hydrolase family 99 |  |  |  |  |  |  |
| TP78757 | TAP42-like family | 0009966 | regulation of signal transduction |  |  |  |  |
| TP141398 | PF13912 |  |  |  |  |  |  |
| TP43151 | PF13912 |  |  |  |  |  |  |
| TP108890 | RNA polymerase II elongation factor ELL | 0006368 | transcription elongation from RNA polymerase II promoter |  |  | 0008023 | transcription elongation factor complex |
| TP15017 | RNA polymerase II elongation factor ELL | 0006368 | transcription elongation from RNA polymerase II promoter |  |  | 0008023 | transcription elongation factor complex |
| TP58247 | Homeobox domain | 0006355 | regulation of transcritpion, DNA-templated | 0043565, 0003700 | sequence-specific DNA binding, sequence-specific DNA binding transcription factor activity |  |  |
| TP15017 | RNA polymerase II elongation factor ELL | 0006368 | transcription elongation from RNA polymerase II promoter |  |  | 0008023 | transcription elongation factor complex |
| TP108890 | RNA polymerase II elongation factor ELL | 0006368 | transcription elongation from RNA polymerase II promoter |  |  | 0008023 | transcription elongation factor complex |
| TP161428 | Mysoin-binding motif of peroxisomes |  |  | 0017022 | myosin binding |  |  |
| TP140088 | TatD related DNase |  |  | 0016888 | endodeoxyribonuclease activity, producing 5'-phosphomonoesters |  |  |
| TP159196 | RhoGAP domain | 0007165 | signal transduction |  |  |  |  |
| TP141187 | Protein kinase domain | 0006468 | 0005524, 0004672 | ATP binding, protein kinase activity |  |  |  |
| TP12046 | C2 domain |  |  | 0005515 | protein binding |  |  |
| TP21957 | Sec7 domain | 0032012 | regulation of ARF protein signal transduction | 0005086 | ARF guanyl-nucleotide exchange factor activity |  |  |
| TP42263 | PX domain |  |  | 0035091 | phosphatidylinositol binding |  |  |
| TP42265 | PX domain |  |  | 0035091 | phosphatidylinositol binding |  |  |
| TP42265 | PX domain |  |  | 0035091 | phosphatidylinositol binding |  |  |
| TP156466 | PX domain |  |  | 0035091 | phosphatidylinositol binding |  |  |
| TP156588 | PX domain |  |  | 0035091 | phosphatidylinositol binding |  |  |
| TP7294 | Sir2 family |  |  | 0070403 | NAD+ binding |  |  |
| TP98498 | Syntaxin |  |  |  |  | 0016020 | membrane |
| TP75186 | Syntaxin |  |  |  |  | 0016020 | membrane |
| TP49081 | RNA recognition motif |  |  | 0003676 | nucleic acid binding |  |  |
| TP148510 | PF13637 |  |  |  |  |  |  |
| TP102142 | PF13637 |  |  |  |  |  |  |
| TP35880 | Resistance to inhibitors of cholinesterase homologue 3 |  |  |  |  |  |  |
| TP8695 | Cyclophilin type peptidyl-prolyl cis-trans isomerase/CLD | 0006457 | protein folding | 0003755 | peptidyl-prolyl cis-trans isomerase activity |  |  |
