# Supplementary material for: Inbreeding and selection shape genomic diversity in captive populations: Implications for the conservation of endangered species
Source: PLoS One. 2017 Apr 19;12(4):e0175996. doi: 10.1371/journal.pone.0175996 (PMC5396937; doi:10.1371/journal.pone.0175996)
Supplement: S2 Table — SNP counts are noted for each replicate protocol, at each generation and the number shared with all previous generations are noted in parentheses. The ‘Unique’ column indicates the number of unique SNPs within the row, whereas “Shared” indicates the number shared within the row. ANOVA indicated that there was no difference between the number of SNPs under selection between protocols (F = 4.8037, df = 2), but there was a significant difference between generations (F = 1.911, df = 2). Our post-hoc Tukey test suggests that the effects of selection increased over time, as generation 19 had significantly more SNPs compared to generation 6 (g19-g6: diff = 67.5, adjusted p = 0.026; g12-g6: diff = 22.5, adjusted p = 0.578; g19-g12: diff = 45.0, adjusted p = 0.168). (DOCX) [file pone.0175996.s002.docx]

S2. Number of nonneutral SNPs (i.e., those that are likely under selection). SNP counts are noted for each replicate protocol, at each generation and the number shared with all previous generations are noted in parentheses. The ‘Unique’ column indicates the number of unique SNPs within the row, whereas “Shared” indicates the number shared within the row. ANOVA indicated that there was no difference between the number of SNPs under selection between protocols (F=4.8037, df=2), but there was a significant difference between generations (F=1.911, df=2). Our post-hoc Tukey test suggests that the effects of selection increased over time, as generation 19 had significantly more SNPs compared to generation 6 (g19-g6: diff=67.5, adjusted p = 0.026; g12-g6: diff=22.5, adjusted p = 0.578; g19-g12: diff=45.0, adjusted p = 0.168).

| **Protocol and population** | | **Gen. 6** | **Gen. 12** | **Gen. 19** | **Unique** | **Shared** |
| --- | --- | --- | --- | --- | --- | --- |
| Random mating | |  |  |  |  |  |
|  | Replicate 1 | 32 | 56 (17) | 130 (40) | 159 | 12 |
|  | Replicate 2 | 97 | 139 (58) | 192 (119) | 241 | 52 |
|  | *Shared within protocol* | 6 | 12 (4) | 28 (8) | 34 | 3 |
| Docility selection | |  |  |  |  |  |
|  | Replicate 1 | 47 | 109 (25) | 121 (45) | 203 | 11 |
|  | Replicate 2 | 129 | -- | -- | 129 | -- |
|  | *Shared within protocol* | 12 | -- | -- | 12 | -- |
| Minimizing mean kinship | |  |  |  |  |  |
|  | Replicate 1 | 40 | 88 (22) | 130 (62) | 170 | 20 |
|  | Replicate 2 | 42 | 43 (14) | 87 (29) | 125 | 12 |
|  | *Shared within protocol* | 1 | 9 (0) | 25 (4) | 31 | 0 |
