# Supplementary material for: Inbreeding and selection shape genomic diversity in captive populations: Implications for the conservation of endangered species
Source: PLoS One. 2017 Apr 19;12(4):e0175996. doi: 10.1371/journal.pone.0175996 (PMC5396937; doi:10.1371/journal.pone.0175996)
Supplement: S3 Table — We used Spearman correlation coefficients (r) and p-values were estimated via 1000 permutations (all p <0.001). (DOCX) [file pone.0175996.s003.docx]

S3. Comparison of SNP estimated genomic diversity (multilocus heterozygosity) to pedigree estimates of genetic diversity (F). We used Spearman correlation coefficients (r) and p-values were estimated via 1000 permutations (all p <0.001).

|  |  | Random | Docility | Mean Kinship | Average |
| --- | --- | --- | --- | --- | --- |
|  | All SNPs | -0.640 | -0.752 | -0.453 | -0.615 |
|  | Nonneutral SNPs | -0.655 | -0.570 | -0.467 | -0.564 |
|  | Neutral SNPs | -0.538 | -0.721 | -0.336 | -0.532 |
|  | **Average** | **-0.611** | **-0.681** | **-0.419** |  |
